# Biochemical and histopathological evaluation of ChAdOx1-S and BBIBP-CorV vaccination in normal and streptozotocin-induced diabetic Wistar rats

**DOI:** 10.1371/journal.pone.0284601

**Published:** 2023-05-04

**Authors:** Mahsa Teymoorzadeh, Negar Daneshfar, Razieh Yazdanparast

**Affiliations:** Institute of Biochemistry and Biophysics, University of Tehran, Tehran, Iran; Gifu University School of Medicine Graduate School of Medicine: Gifu Daigaku Igakubu Daigakuin Igakukei Kenkyuka, JAPAN

## Abstract

Infection with Covid-19 has been associated with some medical complications namely diabetes, thrombosis, and hepatic and renal dysfunction among others. This situation has created some concern about the use of relevant vaccines which might cause similar complications. In that regard, we planned to evaluate the impact of two of the relevant vaccines namely ChAdOx1-S and BBIBP-CorV on some of the blood biochemical factors and also on liver and kidneys functions following the immunization of healthy and streptozotocin-induced diabetic rats. Evaluation of the level of neutralizing antibody among the rats indicated that immunization with ChAdOx1-S induced a higher level of neutralizing antibody among both the healthy and diabetic rats compared to the BBIBP-CorV vaccine. Furthermore, the neutralizing antibody levels against both types of vaccines were significantly lower in diabetic rats than in healthy ones. On the other hand, no alterations were observed in the rats’ sera biochemical factors, coagulation values and histopathological images of the liver and kidneys. Altogether these data besides of confirming the effectiveness of both vaccines, indicate that both vaccines have no hazardous side effects on rats and probably humans though clinical investigations are required to validate our present data.

## Introduction

Coronavirus disease 2019 (Covid-19) is a pandemic disease caused by severe acute respiratory syndrome coronavirus 2 (SARS-CoV-2) [1]. The mortality rate in patients with Covid-19 raises with age and underlying disease, making older people and patients with other diseases more vulnerable than healthy young people [2]. It has been well known that diabetes is a metabolic disorder in which the body’s ability to consume glucose diminishes and as a result, blood glucose increases [3]. This high blood glucose causes some vascular and tissue damages mostly in the kidneys, eyes, and nervous system [4]. It has been estimated that in 2030, 578 million people worldwide will have diabetes, and this number will rise to 700 million by 2045 [5]. Following the outbreak of Covid-19, higher mortality rates became evident among diabetics compared to non-diabetics [6].

During SARS-CoV-2 invasion into host cells, the virus binds to the angiotensin-converting enzyme receptor 2 (ACE2) through its spike protein [7]. ACE2 is expressed on the cell membrane of different organs such as the respiratory system, liver, pancreas, and kidneys [8, 9]. Therefore, these organs are more vulnerable targets for SARS-CoV-2 invasion [10]. Thus, it might worsen the physiological condition of diabetes or ease disease induction [11]. In addition, it has been reported that SARS-CoV-2 binds to ACE2 receptors on the vascular endothelial surface, deregulates the ACE2 cascade and eventually, activates the coagulation system [12]. On the other hand, in diabetic patients, due to increased platelet function and their adhesion to vascular endothelial cells, the risk of clot formation is expected to increase [13]. Consequently, the combination of these two pathways would probably potentiate the risk of blood clot formation among Covid-19 infected diabetic patients.

Moreover, infected people with Covid-19 occasionally show kidney damage [14]. The renal injury could be caused by direct invasion of SARS-CoV-2 into kidney cells or, it could be due to the induction of cytokines following Covid-19 infection [15]. It has frequently been observed that Covid-19 infected people have higher sera levels of liver enzymes such as Alanine aminotransferase (ALT), Alkaline phosphatase (ALP), Aspartate aminotransferase (AST) and also bilirubin with lower albumin level, indicating some level of liver damage [16, 17].

Despite of pandemic perspective of Covid-19, no definitive cure for the disease has been explored and at present, the only option for controlling the disease or reducing the mortality rates is mass vaccination. This approach has been associated with many challenges in various countries mainly because Covid-19 vaccines were developed and produced in a very short time [18, 19]. This might represent the lack of comprehensive information on their possible side effects [20] and thus, brings about the concern of new mutations and the emergence of new strains of the virus by unvaccinated people [21].

BBIBP-CorV (Beijing Institute of Biological Products, Beijing, China) is a β-propiolactone-inactivated, aluminum hydroxide-adjuvanted Covid-19 vaccine which reduces 79% of hospitalization [22]. Common side effects of this vaccine include pain at the injection site and dizziness [23]. ChAdOx1-S (Vaxzevria, AstraZeneca, Oxford, UK) on the other hand, is an adenovirus vector-based vaccine that provides more than 70% immunity in adults [24]. The severity of this vaccine**’**s side effects is usually slight to mild which disappear after a few days [25]. However rare side effects, including thrombosis, have also been reported for this vaccine [26].

Based on some reports, Covid-19 has been associated with diabetes, thrombosis, and hepatic and renal injuries. So, there are public concerns that vaccines, due to the presence of spike proteins, might act similar to Covid-19. Our current non-clinical study is aimed to evaluate whether vaccination by a Covid-19 vector-based vaccine, ChAdOx1-S, and a vaccine containing the inactivated virus, BBIBP-CorV, can induce or worsen diabetes, blood coagulation, or destruct liver and kidneys function.

## Materials and methods

### Materials

Streptozotocin (S0130), xylene (1330-20-7), hematoxylin (H9627) and eosin (HT110116) were purchased from Sigma Aldrich Company (USA). SARS-CoV-2-Neutralizing-Ab Enzyme-linked immunosorbent assay (ELISA) kit was purchased from Pishtaz Teb (Iran). Kits for serum biochemical tests including glucose, triglyceride, cholesterol, albumin, bilirubin, AST, ALT, ALP, urea, uric acid and creatinine were purchased from Darman Faraz Kave (Iran). aPPT (activated partial thromboplastin time) and PT (prothrombin time) kits were purchased from Fisher (USA). Covid-19 vaccines ChAdOx1-S (LOT: ACB6264) and BBIBP-CorV (LOT: B2021092959) were purchased from South Tehran Health Center.

### Animals

Female and male Wistar rats (30 of each) with an initial weight of 180–220 g were randomly divided into six groups, consisting of five female and five male rats. Rats were kept at a temperature between 20–25° C, 30% humidity and 12 hours of light / 12 hours of darkness with access to adequate and healthy food and water.

### Ethics

This project was evaluated by Research Ethics Committees of College of Science-University of Tehran and found to be in accordance with the ethical principles and the national norms and standards of the research ethics committees of college of science of University of Tehran (approval IDs: IR.UT.SCIENCE.REC.1400.018 and IR.UT.SCIENCE.REC.1400.019).

### Induction of diabetes

Wistar rats fasted for 12 hours and then their blood glucose levels were measured using an Accu Chek Activ glucometer (USA) through a cut in their tail [27]. The male and female rats, thirty each with blood glucose levels between 70–90 mg/dl were selected for the experiment and in 15 male and 15 female rats, diabetes was induced by intraperitoneal injection of streptozotocin with a single dose of 50 mg/kg (dissolved in 5 mM citrate buffer, pH 4.5). After 72 hours, the rats were fasted for 12 hours and then their blood glucose levels were measured using an Accu Chek Activ glucometer. A blood glucose level above 250 mg/dl was considered to confirm diabetes [28].

### Experimental design

Group I: normal control rats received 0.5 ml of physiological saline on days 1 and 21.

Group II: normal rats received 0.37 ml of ChAdOx1-S (3.7×1010 viral particles) on days 1 and 21 [29].

Group III: normal rats received 0.5 ml of BBIBP-CorV vaccine (4 μg of inactivated SARS-CoV-2 antigens soluble in aluminum hydroxide adjuvant on days 1 and 21 [30, 31].

Group IV: diabetic rats received 0.5 ml of physiological saline on days 1 and 21.

Group V: diabetic rats received 0.37 ml of ChAdOx1-S (3.7×1010 viral particles) on days 1 and 21.

Group VI: diabetic rats who received 0.5 ml of BBIBP-CorV vaccine (4 μg of inactivated SARS-CoV-2 antigens soluble in aluminum hydroxide adjuvant) on days 1 and 21.

On day 0 of the experiment, one day before the first dose injection, all rats were fasted for 12 hours then, blood samples were taken from their hearts under isoflurane anesthesia for biochemical tests on day 0 [32]. Half of the blood was poured into an empty tube and the other half into a tube containing 3.2% sodium citrate for aPTT and PT. All blood samples were then centrifuged for 15 minutes at 3500 × g and the sera were separated for further biochemical analyses.

On day 49 of the experiment, 28 days after the second vaccine injection, the rats were again fasted for 12 hours, blood was taken from their hearts under isoflurane anesthesia, and each divided into an empty tube and a tube containing 3.2% sodium citrate for biochemical assays. Then rats were euthanized in a gradually filled carbon dioxide (CO2) chamber at a 30% volume per minute displacement rate. Since consciousness is lost when CO2 concentration is between 40%–50%. This protocol will minimize the duration of the animal’s pain while it is conscious [33]. Finally, the liver and kidneys were removed and immediately washed with cold PBS buffer and placed in 10% formalin for 48 hours for histopathological examinations.

### Immunogenicity assay

Neutralizing antibodies levels in rats’ sera were measured using SARS-CoV-2-Neutralizing-Ab ELISA Kit (Pishtaz Teb) according to manufacturer instructions. Briefly, sera samples were added to 96‐well plates coated with spike protein antigens and immediately ACE-2 conjugated to horseradish-peroxidase (HRP) was added to each well. Then, they were incubated for 30 minutes at 37° C. The wells were then washed 5 times with washing buffer. Followed by Chromogen-Substrate addition to the wells and the reaction was stopped by adding the stop solution. Eventually, the absorbance was measured at 450 nm using an ELISA reader.

### Biochemical analyses of rats’ sera

Sera levels of glucose, cholesterol, triglyceride, albumin, bilirubin, AST, ALT, ALP, creatinine, urea and uric acid were measured by Darman Kave kits and aPTT and PT were determined by Thermo Fisher Scientific kits.

### Determination of the bleeding time

Bleeding time (BT) was assessed as the rats were anesthetized and their tails were disinfected using ethanol 70%, and the terminal 1-mm tip of the tail was punctured by a sterile needle. The wound was blotted at time 0 and 15-second time intervals thereafter onto Whatman filter papers till it stopped bleeding. The time when no blood was blotted on the paper was considered the bleeding time [34].

### Histopathological examination

The liver and kidney tissue samples were washed with PBS buffer, then were dehydrated with ethanol and then were placed in xylene and the paraffin, respectively. In the next step, the samples were cut with a microtome to a thickness of 5 micrometers and placed on a slide. After melting the paraffin, tissue sections were rehydrated and then stained with hematoxylin and eosin dyes and examined with a light microscope [35].

### Statistical analysis

The data were analyzed by IBM SPSS Statistics 23 and results are presented as MEAN ± standard error of means (SEM). Statistically significant differences between groups were ascertained using one-way analysis of variance (ANOVA) followed by Tukey’s post-hoc. The differences were considered significant when p ≤ 0.05.

## Result

### Induction of neutralizing antibody

**Fig 1:**
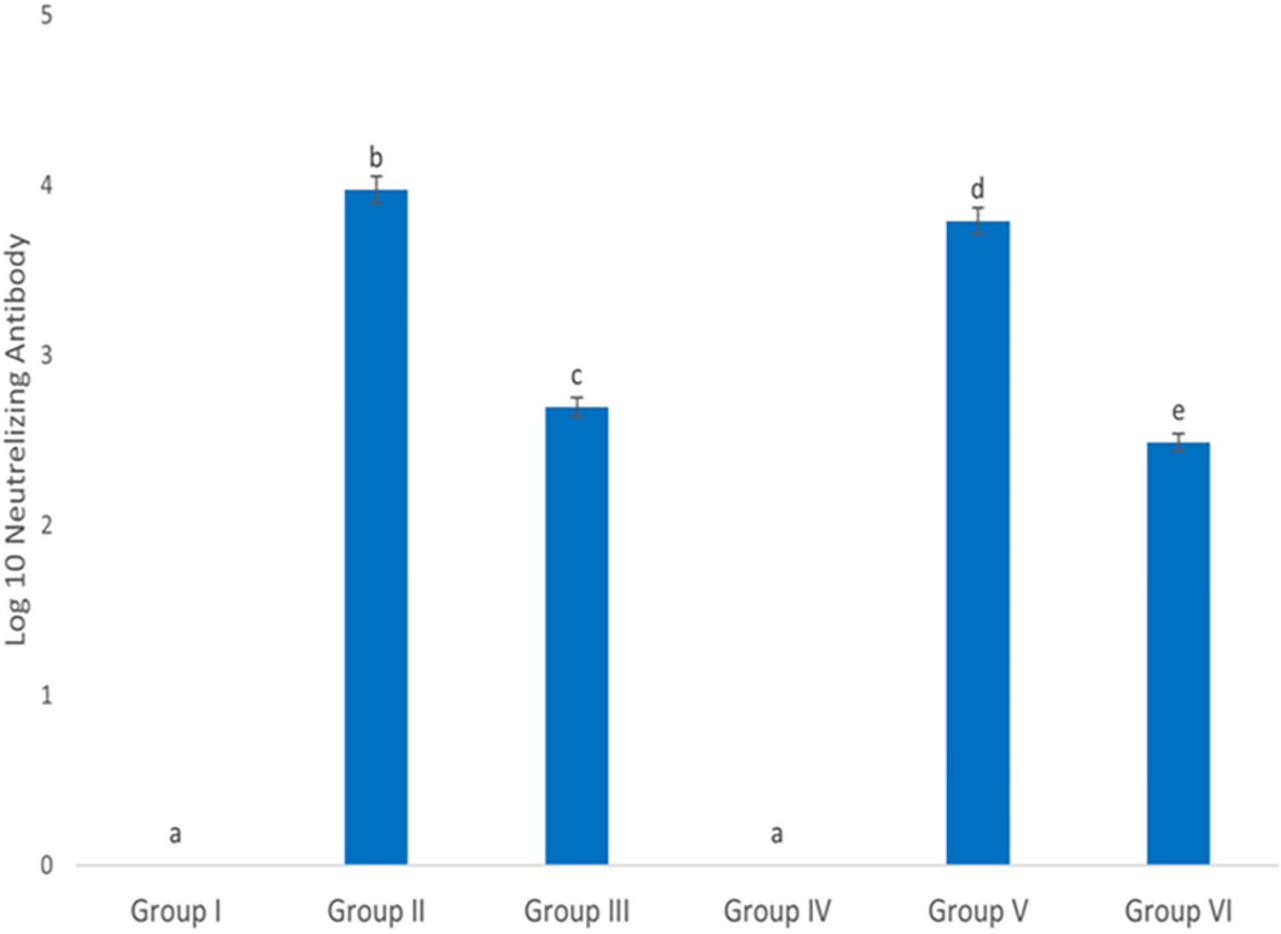
Immunogenicity analyses of the neutralizing antibody response to SARS-CoV-2 spike glycoprotein in vaccinated rats. Each column represents mean ± SEM for 10 rats. Groups’ treatments are presented in the materials and methods section, p < 0.05.

The results demonstrated that injection of each of the ChAdOx1-S or BBIBP-CorV vaccines induced the production of a high level of neutralizing antibodies and all samples from groups II, III, V and VI were above the limit of quantification for the immunogenicity analyses and they were considered seropositive (Fig 1). The neutralizing antibody levels in groups II and V (who had received the ChAdOx1-S vaccine) were impressively high compared to control groups (I and IV, respectively). However, the ChAdOx1-S vaccine induces 5% more neutralizing antibodies in the normal group compared to the diabetic group. The levels of neutralizing antibodies in the sera of groups III and VI (who had received BBIBP-CorV) were significantly lower than the groups receiving the ChAdOx1-S vaccine. Antibody levels in the sera of normal and diabetic groups receiving the BBIBP-CorV vaccine showed a significant difference with each other so this vaccine was able to induce 8% more antibodies in the normal group compared to the diabetic group (Fig 1).

### Rats’ sera levels of glucose, triglyceride and cholesterol

**Fig 2:**
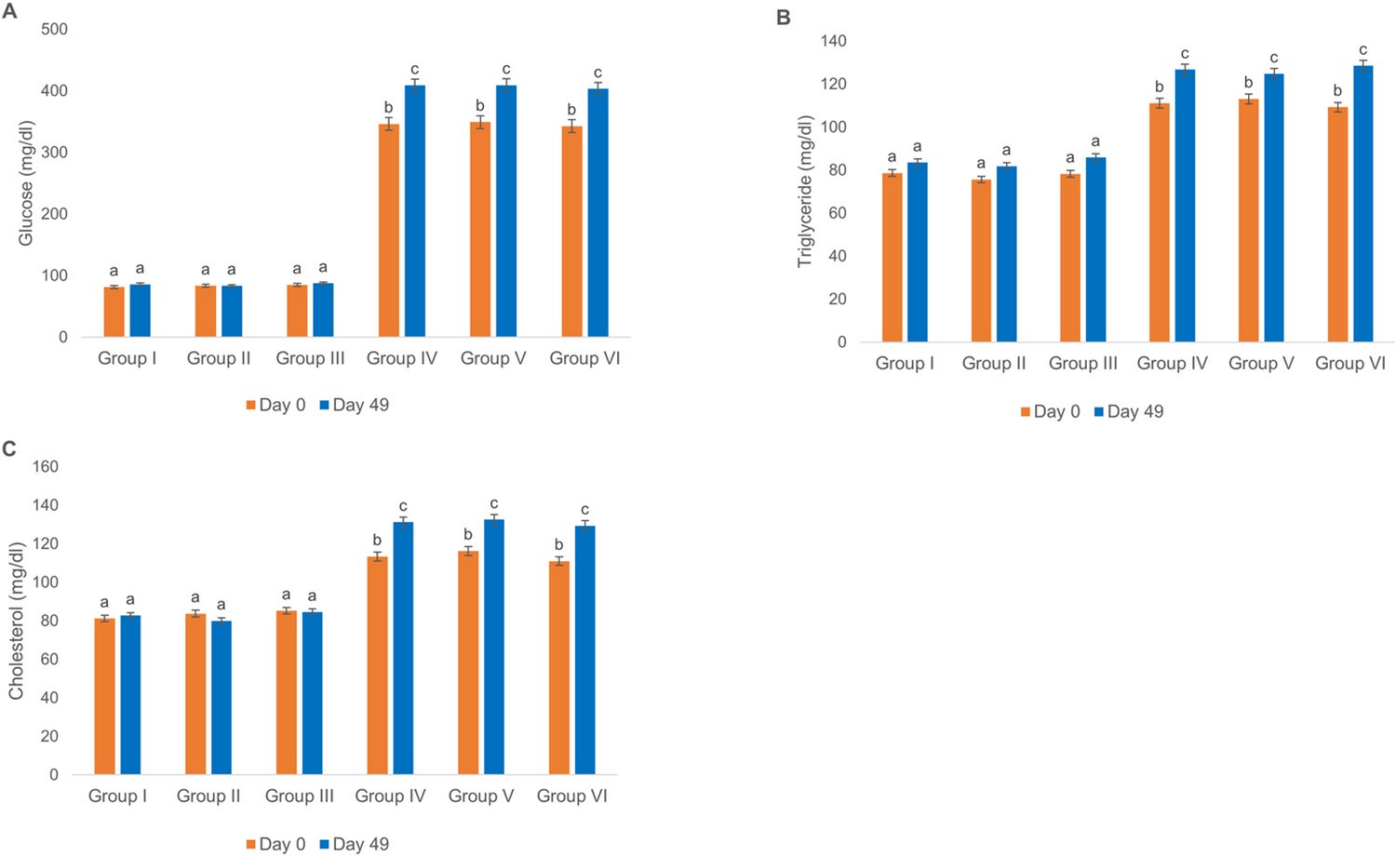
Effect of intramuscular injection of ChAdOx1-S and BBIBP-CorV vaccines on glucose, triglyceride and cholesterol. (A) Glucose, (B) triglyceride and (C) cholesterol levels in sera of normal and diabetic Wistar rats. Groups’ treatments are presented in the materials and methods section, p < 0.05.

Based on the results shown in Fig 2, the sera glucose, triglyceride and cholesterol levels in groups II and III did not show any significant changes compared to group I. Moreover, no significant alterations were observed for the glucose, triglyceride and cholesterol levels of groups V and VI on day 49. However, the sera glucose, triglyceride and cholesterol levels of rats in all diabetic groups elevated significantly on day 49 when compared to day 0.

### Biochemical and histopathological evaluation of rats’ livers following vaccination with oChAdOx1-S and BBIBP-CorV

**Fig 3:**
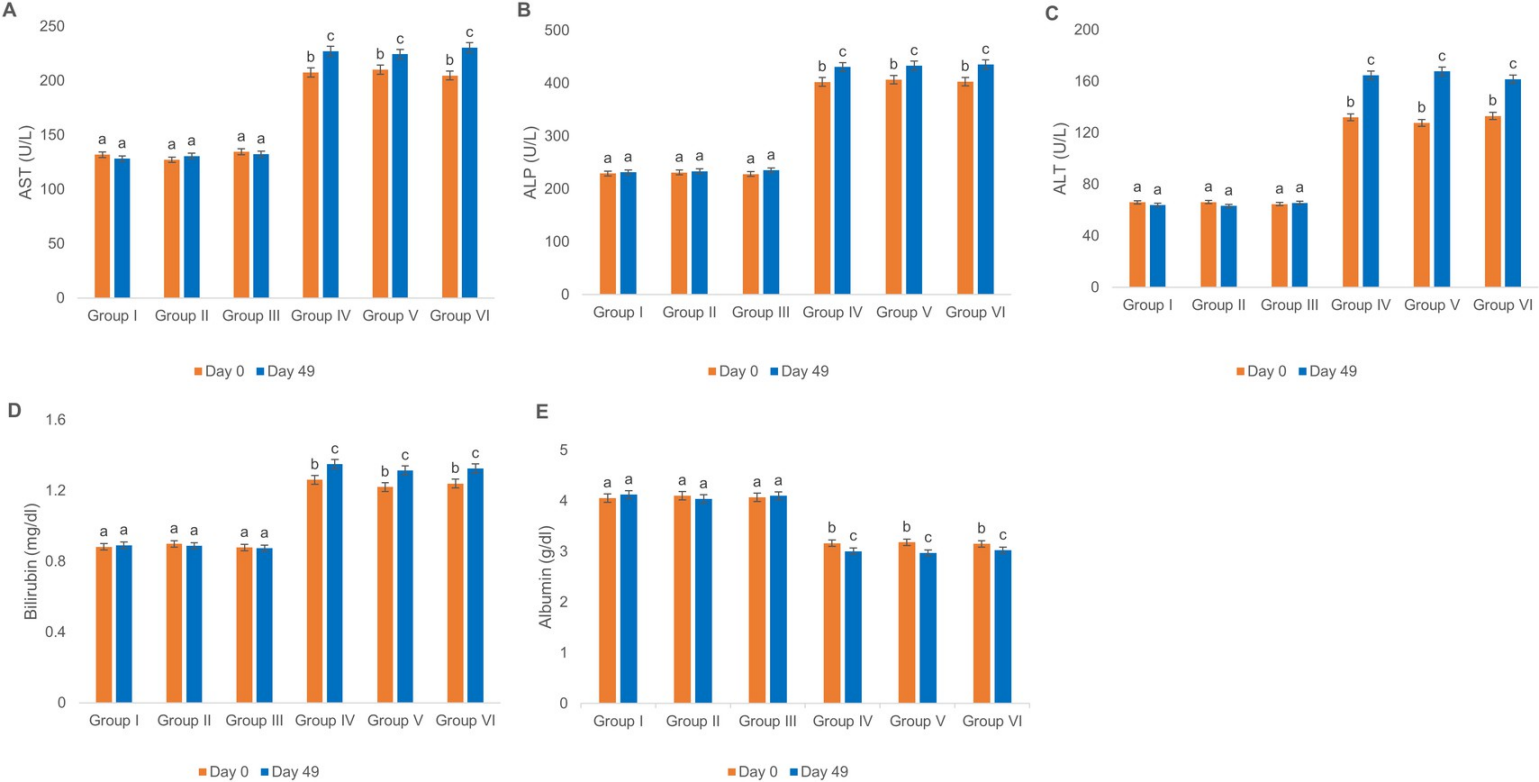
Effect of ChAdOx1-S and BBIBP-CorV vaccination on sera biochemical factors related to liver function. (A) ALT, (B) AST, (C) ALP, (D) bilirubin and (E) albumin levels in sera of normal and diabetic Wistar rats. Groups’ treatments are presented in the materials and methods section. The control groups received the vehicle (physiological saline), p < 0.05.

As shown in Fig 3, injection of ChAdOx1-S and BBIBP-CorV vaccines did not alter sera levels of AST, ALT, ALP, bilirubin and albumin of group II and III relative to group I. Besides, the present results showed that in diabetic rats on day 0, the sera levels of these liver factors in group V and VI were not significantly different from group IV. Moreover, after intramuscular injection of either ChAdOx1-S or BBIBP-CorV vaccines on day 49 of the experiment, statistical changes were not observed in sera AST, ALT, ALP, bilirubin and albumin levels as compared to group IV. However, in groups IV, V and VI a significant elevation in the sera levels of AST, ALT, ALP and bilirubin levels and a notable decrease in the sera levels of albumin were observed on day 49 when compared to day 0.

**Fig 4:**
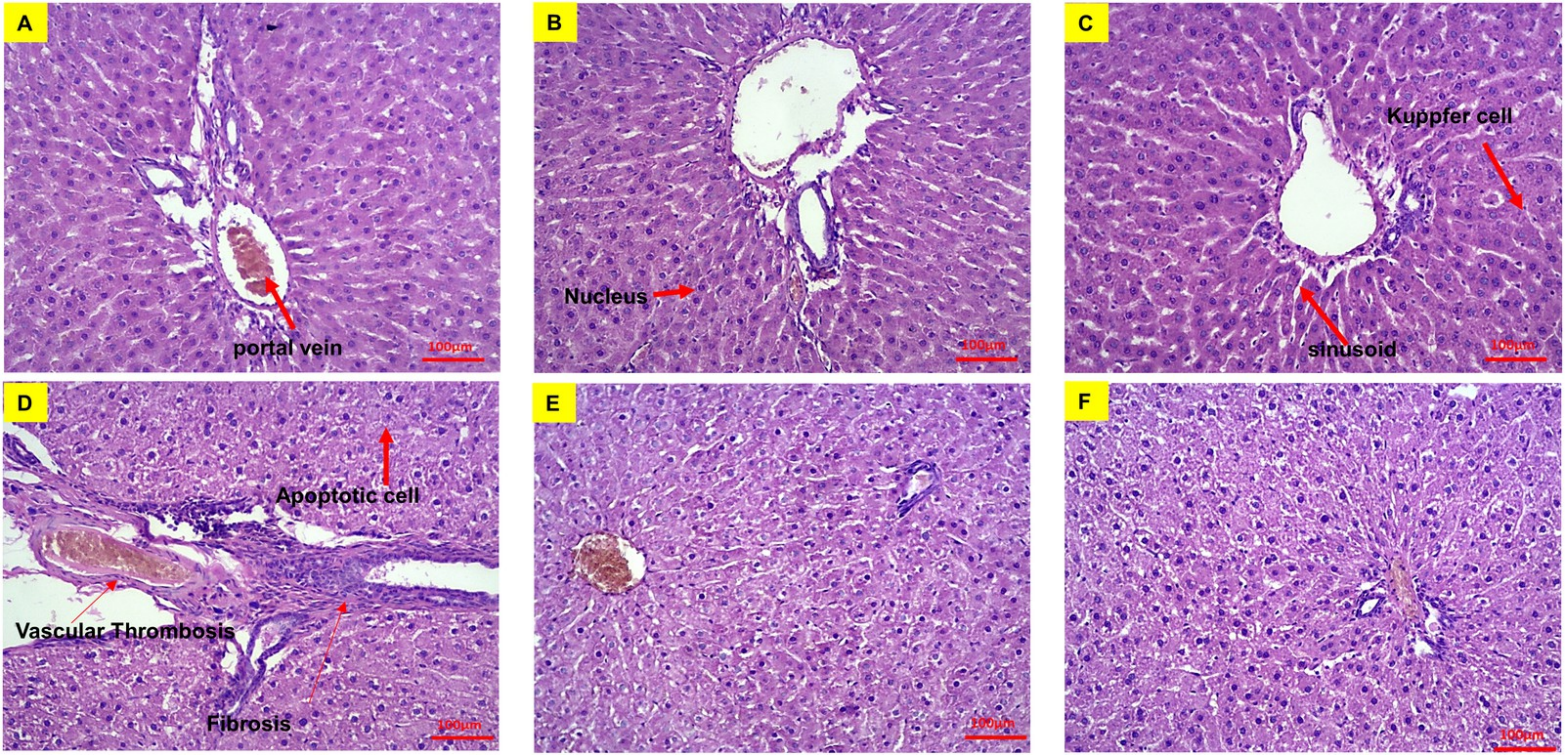
Cross-sectional images of rat liver tissues stained with hematoxylin and eosin dyes. (A) Group I, (B) group II, (C) group III, (D) group IV, (E) group V and (F) and group VI. In these images the nuclei are purple and the cytoplasm is pink. Bar represents 100 μm.

Also, histopathological examinations of the liver samples pointed out that hepatic cells had a normal appearance in non-diabetic groups (groups I, II and III). In the space between the lobules, a central vein with a normal appearance was observed. Hepatocytes without nuclei and with a similar appearance to apoptotic cells were rarely observed (Fig 4). However, images related to groups IV, V and VI showed a few Hepatocytes without nuclei and with apoptotic appearance (Fig 4).

**Fig 5:**
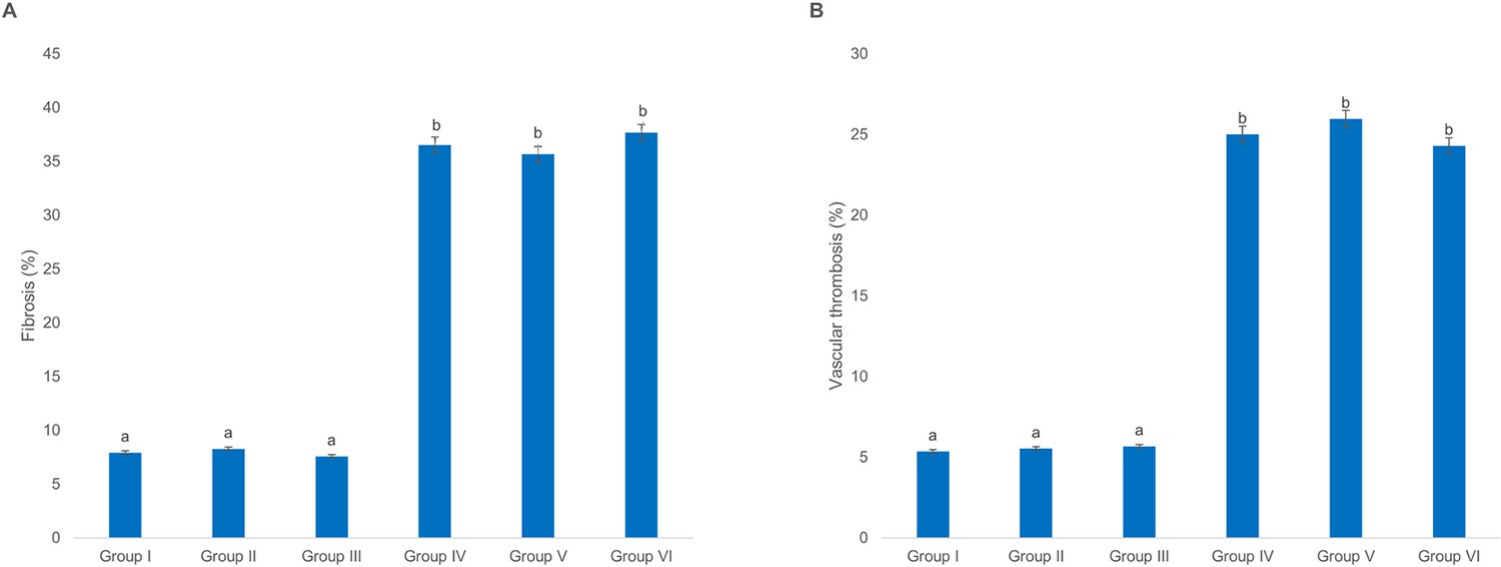
Rats’ liver histopathological changes following vaccination with ChAdOx1-S and BBIBP-CorV. The percentages of (A) liver fibrosis and (B) vascular thrombosis in normal and diabetic Wistar rats. Each column represents mean ± SEM for 10 rats. Groups’ treatments are presented in the materials and methods section, p < 0.05.

Despite Sonzogni et al. report concerning the adverse effect of Covid-19 infection on the liver [36], no changes were observed in the percentage of liver fibrosis and vascular thrombosis in groups II and III and groups V and VI when compared to their relevant control groups. However, a significant increase was detected in the percentage of fibrosis and vascular thrombosis in the livers of all diabetic groups compared to their relevant normal ones (Fig 5).

### Biochemical and histopathological evaluation of rats’ kidneys following vaccination with oChAdOx1-S and BBIBP-CorV

**Fig 6:**
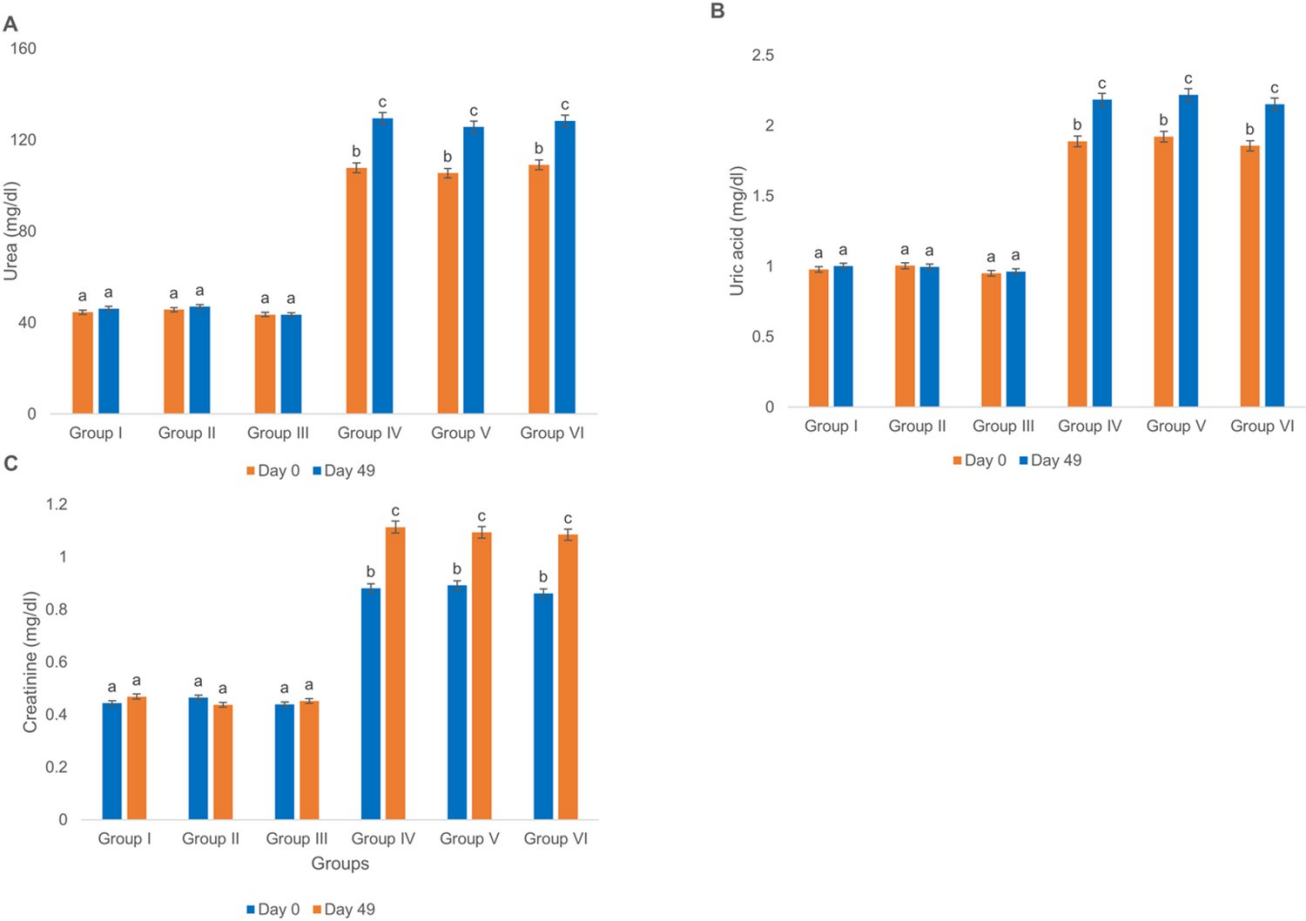
Effect of ChAdOx1-S and BBIBP-CorV vaccination on sera biochemical factors related to kidney function. (A) Urea, (B) uric acid and (C) creatinine levels in sera of normal and diabetic Wistar rats. Groups’ treatments are presented in the materials and methods section, p < 0.05.

Intramuscular injection of ChAdOx1-S and BBIBP-CorV vaccines produced no alteration in the mean sera of biochemical markers related to renal function including urea, uric acid and creatinine in groups II and III as compared to group I. Furthermore, in diabetic groups, sera urea, uric acid and creatinine levels of groups V and VI exhibited statistically non-significant changes when compared to group IV on day 0. Also, 28 days after the second dose injection, these levels did not show any considerable difference as compared to group IV. However, similar to the case of sera biochemical markers related to liver function, the rats of all diabetic groups exhibited a statistically significant increase in the sera urea, uric acid and creatinine levels on day 49 as compared to those on day 0 (Fig 6).

**Fig 7:**
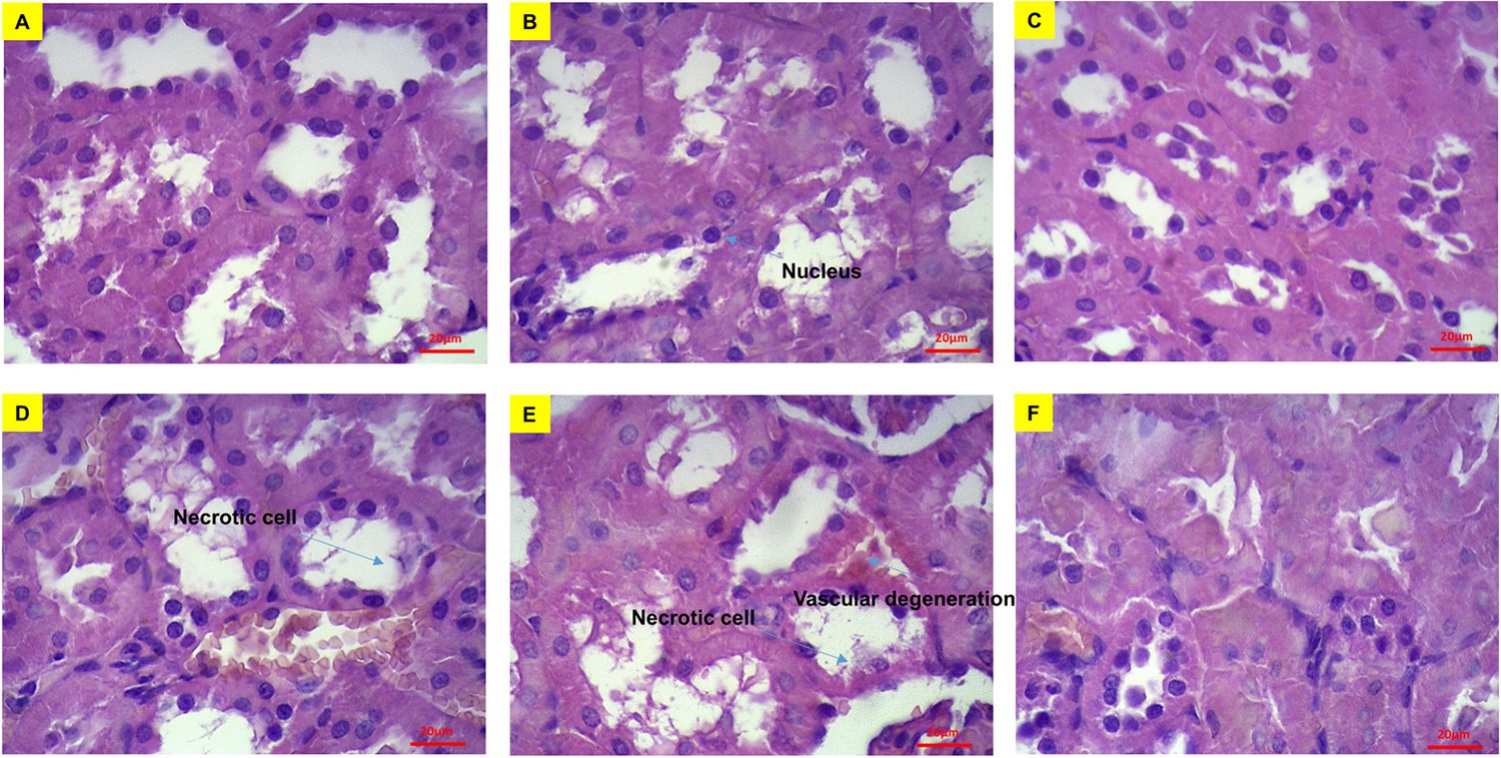
Cross-sectional images of rats’ kidney tissue stained with hematoxylin and eosin dyes. (A) Group I, (B) group II, (C) group III, (D) group IV, (E) group V and (F) and group VI. In these images the nuclei are purple and the cytoplasm is pink. Bar represents 20 μm.

Based on the images presented in Fig 7, the renal cortex possessed a normal shape and there were no deformations in epithelial cells of distal and proximal convoluted tubules in normal groups. No histopathological alteration was observed in the kidney of rats of Group II and III as compared to group I. The size of glomeruli in all diabetic rats’ kidneys diminished when compared to normal tissue and bleeding was observed inside the Bowman’s capsule. The percentage of lymphocytes in renal tissue increased in all diabetic groups when compared to their relevant normal groups and the death of epithelial cells of the Bowman’s capsule wall was observed in the glomeruli. Also, in diabetic groups, the secretion of interstitial lymphocytes was much higher than control normal group.

**Fig 8:**
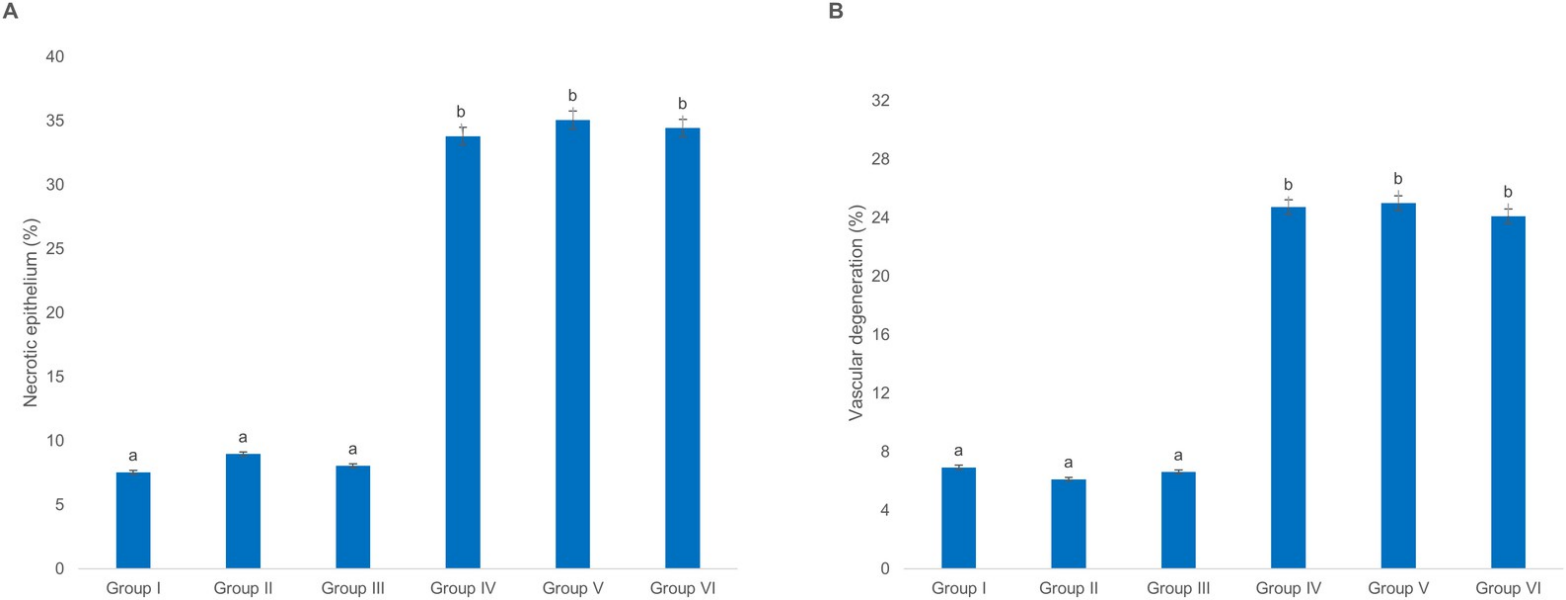
Rats’ kidneys histopathological changes following vaccination with ChAdOx1-S and BBIBP-CorV. (A) The percentages of renal necrotic epithelium and (B) vascular degeneration of normal and diabetic Wistar rats. Each column represents mean ± SEM for 10 rats. Groups’ treatments are presented in the materials and methods section, p < 0.05.

The percentage of the necrotic epithelium and vascular degeneration as two reported kidney damages following Covid-19 infection [37], in groups II and III and groups V and VI were not different from their relevant control groups, but all diabetic groups exhibited an elevation in the percentage of renal necrotic epithelium and vascular degeneration when compared to their relative normal groups (Fig 8).

### The effect of ChAdOx1-S and BBIBP-CorV vaccines on coagulation values in rats’ sera

**Fig 9:**
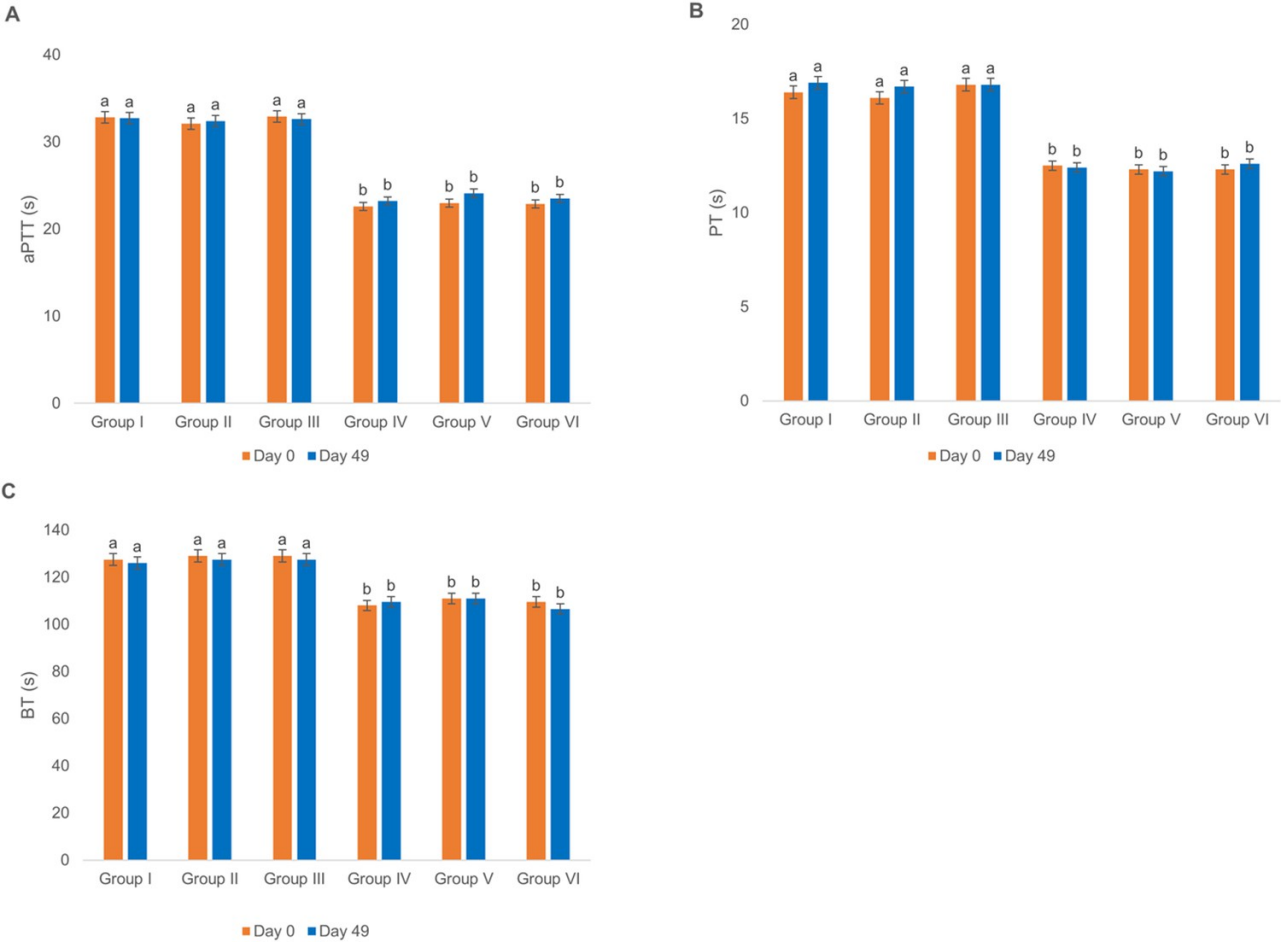
Effect of ChAdOx1-S and BBIBP-CorV vaccination on sera coagulation values. (A) aPTT, (B) PT and (C) BT values in sera of normal and diabetic Wistar rats. Groups’ treatments are presented in the materials and methods section. The control groups received the vehicle (physiological saline), p < 0.05.

The aPTT, PT and BT values were measured to evaluate the effects of ChAdOx1-S and BBIBP-CorV vaccines on the rats’ coagulation system. Fig 9 shows that the injection of ChAdOx1-S and BBIBP-CorV vaccines into rats of groups II and III did not lead to variation in the aPTT, PT and BT values as compared to that of group I. similar results were obtained for the diabetic vaccine-treated rats (Fig 9).

## Discussion

Effective immunization with minimal side effects following vaccination is one of the main concerns for all people [38], especially those with underlying disorders such as diabetes [39]. On that ground, we performed a non-clinical evaluation of the effect of ChAdOx1-S and BBIBP-CorV vaccines on normal and diabetic rat models to evaluate the safety and efficacy of these vaccines. Our data indicated no vaccine-related deaths, unpredicted severe side effects and significant alteration in the mean body weights or food and water consumption during the experiment. Moreover, no variation in the size and color of tissues such as the liver and kidneys were observed with the naked eye among all groups. In line with the findings of Shrotri and colleagues, the induced antibody levels among the diabetic groups were lower (by about 4%) than the normal ones following immunization with the vaccines used in our investigation [40]. In addition, our data indicated that immunization with the ChAdOx1-S vaccine produced more antibodies than vaccination with the BBIBP-CorV vaccine.

Our histopathological data did not show any damage to liver and kidney tissues following the injection of the vaccines. These data were confirmed by some of the measured biochemical factors (Figs 1–4). However, our observations were not in line with some case reports on liver and kidney damage following Covid-19 vaccination [41, 42]. These variations might be due to the differences among the experimental species, vaccine types (most reports on liver and kidney damages, following vaccination, are related to the Pfizer-BioNTech vaccine) or the size of statistical populations (the side effects might be seen in bigger statistical populations) [37–42]. Coagulation parameters such as aPTT, PT and BT represent the functional status of the blood coagulation condition [43]. There have been some reports that Covid-19 vaccines (especially the ChAdOx1-S vaccine) are associated with thrombosis [26], but our evaluation of aPTT, PT and BT among normal and diabetic rats did not show any variation that was expected. Although the statistical population is too small to draw definitive conclusions in this case, however, these data could show that the risk of clot formation following injection of Covid-19 vaccines among the diabetic groups was not higher than the normal groups.

Due to the lack of a definitive cure for Covid-19, it seems that the only way to defeat this epidemy is immunization against Covid-19 [44]. To reach this goal sufficient and reliable data supporting the safety of the vaccines should be provided to build up global confidence for those who avoid Covid-19 vaccination [45]. Therefore, there is an urgent demand for more comprehensive studies on the probable side effects of the Covid-19 vaccines.

### Conclusion

The results of this study present supportive evidence of the effectiveness and safety of two Covid-19 vaccines on some of the biochemical variables and clotting factors. Although these data cannot be extrapolated directly from Wistar rats to humans, however, they provide the ground for expanding the investigation to the clinical levels.
